# Objective Perfusion Assessment in Gracilis Muscle Interposition—A Novel Software-Based Approach to Indocyanine Green Derived Near-Infrared Fluorescence in Reconstructive Surgery

**DOI:** 10.3390/life12020278

**Published:** 2022-02-13

**Authors:** Leonard A. Lobbes, Richelle J. M. Hoveling, Leonard R. Schmidt, Susanne Berns, Benjamin Weixler

**Affiliations:** 1 Department of General and Visceral Surgery, Hindenburgdamm 30, Charité—Universitätsmedizin Berlin, corporate member of Freie Universität Berlin and Humboldt-Universität zu Berlin, 12203 Berlin, Germany; leonard.schmidt@charite.de (L.R.S.); susanne.berns@charite.de (S.B.); benjamin.weixler@charite.de (B.W.); 2 Quest Medical Imaging, 1775 PW Middenmeer, The Netherlands; richelle.hoveling@quest-innovations.com

**Keywords:** indocyanine green, ICG, near-infrared fluorescence, perfusion assessment, gracilis muscle interposition, GMI, rectovaginal fistula, pouchovaginal fistula, inflammatory bowel disease, IBD

## Abstract

Background: Gracilis muscle interposition (GMI) is an established treatment option for complex perineal fistulas and reconstruction. The outcome is limited by complications such as necrosis, impaired wound healing and fistula persistence or recurrence. Quantifiable methods of assessing muscle flap perfusion intraoperatively are lacking. This study evaluates a novel and objective software-based assessment of indocyanine green near-infrared fluorescence (ICG-NIRF) in GMI. Methods: Intraoperative ICG-NIRF visualization data of five patients with inflammatory bowel disease (IBD) undergoing GMI for perineal fistula and reconstruction were analyzed retrospectively. A new software was utilized to generate perfusion curves for the specific regions of interest (ROIs) of each GMI by depicting the fluorescence intensity over time. Additionally, a pixel-to-pixel and perfusion zone analysis were performed. The findings were correlated with the clinical outcome. Results: Four patients underwent GMI without postoperative complications within 3 months. The novel perfusion indicators identified here (shape of the perfusion curve, maximum slope value, distribution and range) indicated adequate perfusion. In one patient, GMI failed. In this case, the perfusion indicators suggested impaired perfusion. Conclusions: We present a novel, software-based approach for ICG-NIRF perfusion assessment, identifying previously unknown objective indicators of muscle flap perfusion. Ready for intraoperative real-time use, this method has considerable potential to optimize GMI surgery in the future.

## 1. Introduction

Gracilis muscle interposition (GMI) is an established treatment option for rectovaginal (RVF), pouch–vaginal (PVF) and recto–urethral (RUF) fistulas, as well as for perineal defects, either primarily or when other procedures have failed [1,2,3,4,5].

The reported success rates of GMI range between 40–90% and tend to be higher with additional local procedures, such as advancement flaps [6,7,8,9,10]. For patients with Crohn’s disease, which, along with ulcerative colitis (UC) and previous radiation therapy, is a risk factor for unsuccessful fistula repair, the success rate of GMI has been reported to be as high as 92% [11,12,13].

However, postoperative complications include muscle necrosis, impaired wound healing, harvest site infection, incontinence, and fistula recurrence [1,6]. Reported rates of fistula recurrence range between 12–15% and are higher for patients with IBD [13,14].

The technical aim of GMI is to place healthy, well-vascularized muscle tissue in the space between the former fistula openings after fistula excision and closure from both sides [15]. The identification and meticulous preparation of the proximal neurovascular bundle carrying the main blood supply of the gracilis muscle is crucial for the procedure [15,16]. Thus, assessing muscle perfusion after complete mobilization, and before final placement, is decisive.

To date, intraoperative perfusion assessment relies on subjective parameters, such as tissue color and the presence of a palpable pulse. These may be affected by a multitude of factors, such as the surgical site and individual experience of the surgeon.

Quantifiable methods of perfusion assessment are still lacking. One method showing promising results is indocyanine green derived near-infrared fluorescence (ICG-NIRF) [17,18,19]. As intravenously administered indocyanine green (ICG) initially remains in the intravascular compartment due to its plasma-binding properties, its near-infrared fluorescence signal is proportional to perfusion [20,21].

However, standardized and objective criteria for ICG-NIRF assessment have not been uniformly established, causing its interpretation to remain subjective in many cases. Furthermore, an ICG-NIRF perfusion assessment has not been previously reported for GMI in correlation with the clinical outcome.

The aim of this study was to evaluate a novel and objective software-based method for ICG-NIRF perfusion assessment in GMI.

## 2. Materials and Methods

In a single-center retrospective study, all patients that had undergone GMI with intraoperative ICG-NIRF imaging at our institution (Charité University Hospital Berlin, Campus Benjamin Franklin, a tertiary colorectal and IBD referral center) from October 2019 to October 2020 were included. All procedures were performed by a single surgeon.

The indications for GMI were RVF, PVF and a persisting perineal defect. Underlying conditions were Crohn’s disease, ulcerative colitis or indeterminate colitis. All patients gave written informed consent for the procedure and ICG-NIRF visualization.

Ethical approval of the study was obtained from The Charité University Hospital Ethics Committee (EA4/029/21, Ethikkommission der Charité—Universitätsmedizin Berlin).

### 2.1. Surgical Technique

The procedure began with fistula tract identification by probing, followed by a perineal incision. The fistula was then debrided, resecting the components. After sufficient irrigation, the fistula openings were closed by interrupted sutures. This was followed by preparation of the remaining perineal space.

Access to the gracilis muscle was gained via two longitudinal incisions on the medial aspect of the thigh, one proximal and one distal. The muscle was mobilized by careful preparation to avoid impairment of the proximal neurovascular bundle. Smaller vessels and collaterals were ligated. After complete mobilization, the gracilis tendon was transected above its insertion, resulting in a completely mobile muscle. This was followed by the preparation of a subcutaneous tunnel from the medial thigh to the perineal incision. The mobilized gracilis muscle was then transferred through this tunnel, allowing its distal segment to be placed in the space between the divided fistula components in a tension-free manner and to be secured by suturing. The perineal wound was closed primarily by suturing. In two cases, fecal diversion by laparoscopic ileostomy was performed additionally to GMI. All other patients had already undergone fecal diversion prior to the procedure.

### 2.2. Intraoperative ICG-NIRF Perfusion Visualization

**Figure 1:**
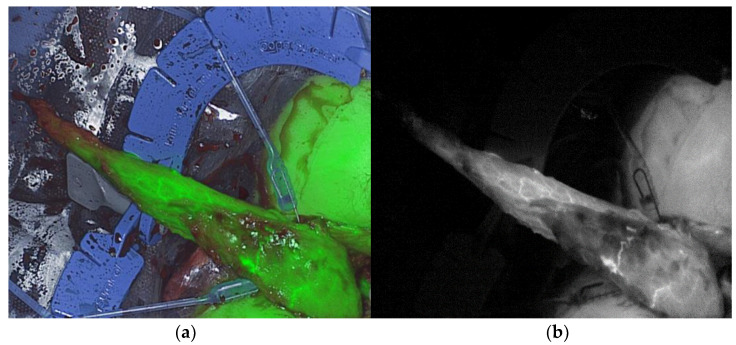
Real-time intraoperative ICG-NIRF perfusion visualization of the gracilis muscle after mobilization and transfer to the perineal region, before final placement: (**a**) overlay mode of color and near-infrared real-time visualization; (**b**) near-infrared fluorescence mode real-time visualization.

Real-time intraoperative ICG-NIRF perfusion visualization of the gracilis muscle was performed after mobilization and transfer to the perineal region, before final placement, with the Quest Spectrum^®^ Fluorescence Imaging Platform (Quest Medical Imaging, Middenmeer, The Netherlands) equipped for open surgery (Figure 1).

ICG (VerDye, Diagnostic Green GmbH, Aschheim Germany, 25 mg vials) was dissolved in 5 mL sterile water to yield a 5 mg/mL concentration. It was then administered intravenously as a bolus of 1 mL (5 mg). Each visualization was video recorded to enable postoperative assessment. Clinical and technical data during visualization were collected. In particular, hemodynamics and the distance of the camera to the imaging site were recorded in detail and standardized as far as possible in the clinical set-up. The time to visual detection of a fluorescence signal (time to fluorescence signal) and subjective perception of signal strength were determined (0 = no signal, 1 = detectable signal, 2 = strong, homogenous signal).

### 2.3. Postoperative Follow-Up

Follow-up consisted of a digital rectal and vaginal exam, vaginoscopy and rectoscopy or pouchoscopy, and was initially conducted within 6 weeks after surgery. A second follow-up was conducted 3 months after surgery or if symptoms occurred.

Clinical data were collected from all patients on postoperative complications of GMI within 3 months. GMI failure was defined as the occurrence of one of the following: persistence or recurrence of the preoperative fistula; required resection of the interposed gracilis muscle due to necrosis or wound site infection; postoperative incontinence.

If none of these complications occurred, the outcome was categorized as successful GMI.

### 2.4. Postoperative Software-Based Assessment of ICG-NIRF Perfusion Visualization Data

The recordings of the ICG-NIRF perfusion visualization were analyzed using a novel perfusion assessment software (Quest Research Framework, Quest Medical Imaging, Middenmeer, The Netherlands).

**Figure 2:**
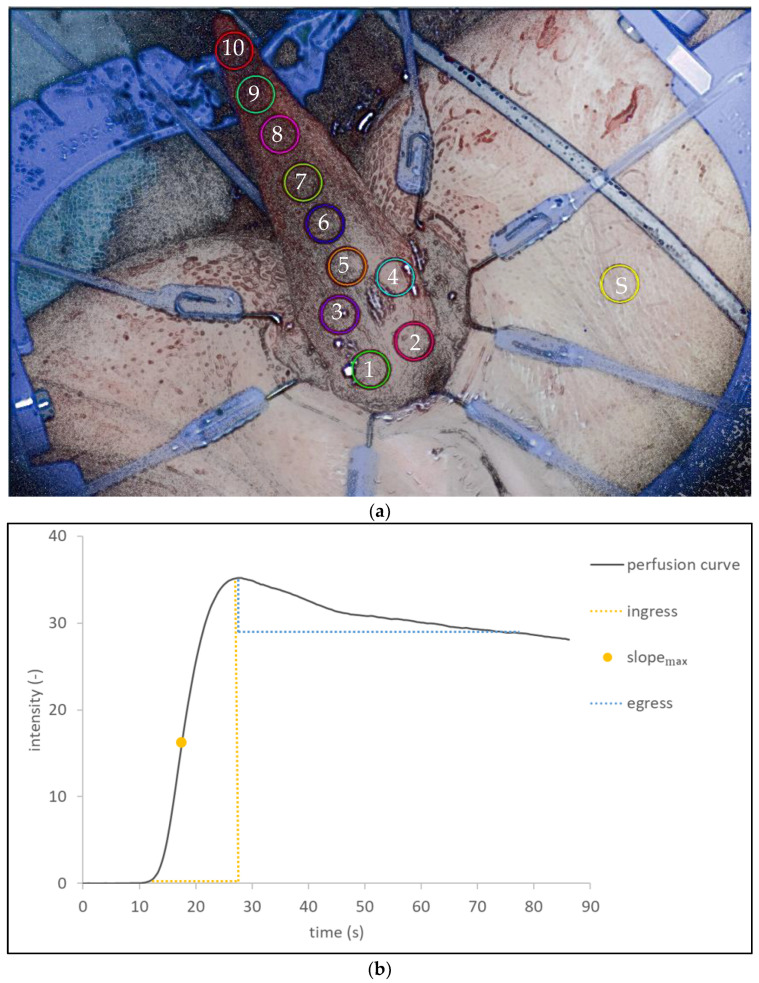
(**a**) Standardized regions of interest (ROIs) were determined in each visualization recording, covering all areas of the mobilized gracilis muscle from the proximal base to the distal tip (numbered 1–10), including an ROI on the skin as a positive control. (**b**) For each ROI, the change in fluorescence intensity (*y*-axis) was visualized over time (*x*-axis), resulting in a perfusion curve. Recurring characteristics of perfusion curves were identified: the ingress phase, defined by a positive slope of the perfusion curve, represents the inflow of blood. The point at which the slope of the perfusion curve has reached its maximum value is defined as slope_max_ and describes the maximum rate of blood inflow. The peak of the perfusion curve (maximum intensity) is defined as the egress phase and represents the outflow of blood. (s) = seconds.

Standardized regions of interest (ROIs) were determined in each visualization recording, covering all areas of the mobilized gracilis muscle from the proximal base to the distal tip and numbered from 1 to 10, including an ROI on the skin as a positive control to indicate sufficient visualization, the influence of confounders and reproducibility (Figure 2a). The change in fluorescence intensity over time was then visualized for each ROI (Figure 2b). In this graph, the ingress and egress region of the perfusion process of the ICG bolus through the tissue is visualized. For the ingress (positive slope) phase of the curve, the maximum slope was calculated.

Additionally, fluorescence intensity over time was assessed in an automated pixel-to-pixel analysis, depicting maximum slope per value per pixel in a color-coded and quantitative manner.

The findings were then correlated with the clinical outcome of each GMI.

## 3. Results

Clinical characteristics (Table 1) and technical data during visualization (Table 2) confirmed, in detail, the standardization of the intraoperative method applied in all patients.

Patient 1, a 52-year-old woman with indeterminate colitis, presented with RVF recurrence after repeated advancement flap. A laparoscopic loop ileostomy and GMI were performed. The intraoperative ICG-NIRF perfusion visualization showed a sufficient perfusion of the mobilized gracilis muscle. The follow-up showed no sign of persistence or recurrence. The perineal and harvest site wounds had healed primarily. At the second follow-up, the successful fistula repair was confirmed (Table 1).

Patient 2, a 29-year-old woman with Crohn’s disease, underwent open proctolocectomy and end ileostomy for severe refractory Crohn’s colitis. Ten days after surgery, the rectoscopy revealed rectal stump leak and vaginal air insufflation. Primary vaginal fistula closure was followed by endorectal vacuum therapy for 16 days. The follow-up revealed RVF recurrence. A month later, GMI was performed. The intraoperative ICG-NIRF perfusion visualization showed sufficient perfusion. The follow-up showed no sign of fistula persistence or recurrence. The perineal and harvest site wounds were healing primarily. The second follow-up confirmed successful fistula repair and complete wound healing.

Patient 3, a 42-year-old woman with Crohn’s disease, had previously undergone colectomy with end ileostomy and a low anterior resection of the rectum with closure of the rectal stump. This patient presented with chronic leak of the rectal stump and underwent a transanal completion mucosectomy with primary endoluminal vacuum therapy. After prolonged endoluminal vacuum therapy, a large perineal wound defect remained. Subsequently, a transanal sphincterectomy with closure of the perineal wound by GMI was performed. The intraoperative ICG-NIRF perfusion visualization showed sufficient perfusion. The initial follow-up showed sufficient perineal repair 11 days after surgery. At the second follow-up 3 months after surgery, the perineal wound had healed completely.

Patient 4, a 52-year-old woman with UC presented with repeated PVF recurrence after ileostomy reversal, having previously undergone redo ileal pouch for PVF. Laparoscopic loop ileostomy and GMI were performed. Intraoperatively, due to possible proximal pedicle impairment, an ICG-NIRF perfusion visualization was performed twice: directly after mobilization before tunneling to the perineal region, and again before final placement (at the same time point as in the previous cases). As the second visualization occurred only 16 min after the first, an increased dose of 15 mg of ICG was used to reduce the effect of background fluorescence. Although the ICG-NIRF perfusion visualization showed a lower signal at the tip of the interposed gracilis muscle, the overall fluorescence signal indicated sufficient overall perfusion on subjective interpretation.

At the first follow-up visit, there was no clinical sign of muscle necrosis or fistula persistence. The second follow-up showed a perineal wound dehiscence in secondary healing, with no evidence of GMI necrosis or fistula. At the third follow-up visit 6 weeks after surgery, pouchoscopy revealed vaginal air insufflation, highly suggestive for PVF persistence. Successful redo GMI from the opposite side was performed thereafter.

Patient 5, a 36-year-old woman with indeterminate colitis, presented with PVF after ileostomy reversal 6 months after having undergone a restorative proctocolectomy with ileal J-pouch. A primary fistula closure and redo ileostomy were performed, followed by GMI 2 months later. The ICG-NIRF perfusion visualization showed sufficient perfusion. The follow-up revealed a complete primary healing of the perineal and harvest site wounds and no sign of fistula persistence or recurrence. A second follow-up confirmed complete healing and successful fistula repair.

**Figure 3:**
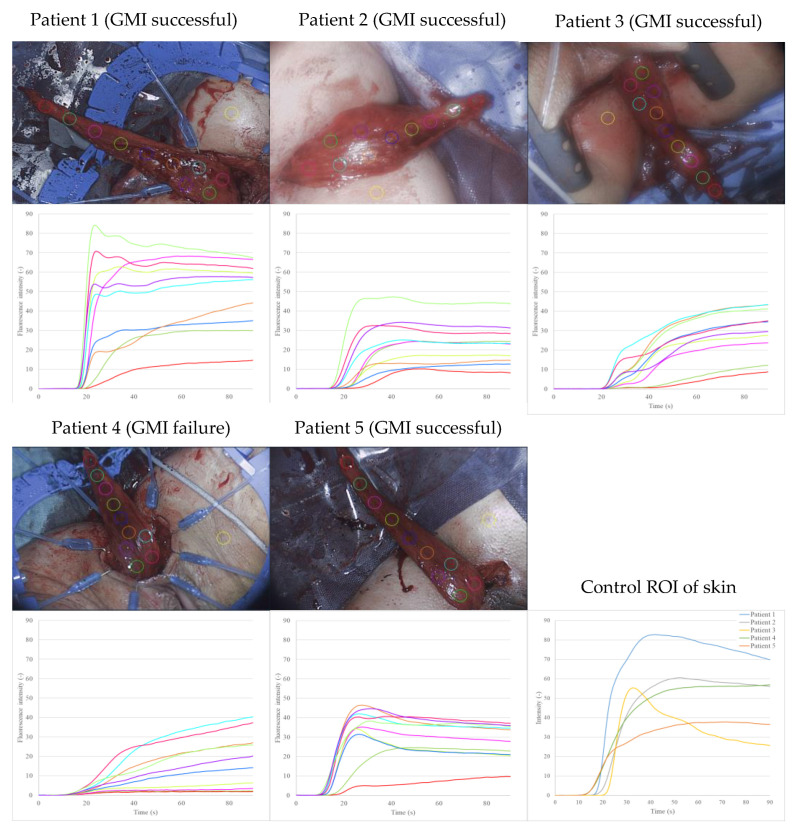
The visualization of fluorescence intensity over time for each predefined ROI (top image: picture per patient) resulted in perfusion curves (bottom image: graph per patient), representing the inflow and outflow of blood in the gracilis muscle. The fluorescence intensity was higher for ROIs near the base of the mobilized gracilis muscle and decreases distally. In general, the shape of the perfusion curves followed a pattern: a steep, positive slope (ingress) followed by flattening out and negative slope (egress). This was particularly apparent in patients 1, 2 and 5 (GMI successful). Patient 3 (successful GMI) showed an ingress twice, with a more gradual flattening out of the curves. In contrast, the perfusion curves of patient 4 (GMI failure) did not show flattening out or a negative slope across all ROIs of the gracilis muscle and lacked a positive slope in the distal ROIs, whilst having low overall fluorescence intensity.

The retrospective visualization of fluorescence intensity over time as a graph for each predefined ROI resulted in perfusion curves, representing the inflow and outflow of blood in the gracilis muscle (Figure 3). The fluorescence intensity was higher for ROIs near the base of the mobilized gracilis muscle and decreased towards the tip.

In general, the shape of the perfusion curves followed a pattern: a steep, positive slope (ingress) was followed by a flattening out and negative slope (egress) of the curve. This was particularly apparent in patients 1, 2 and 5 (successful GMI). The perfusion curves of patient 3 (successful GMI) showed an ingress twice, with a more gradual flattening of the curves. In contrast, the perfusion curves of patient 4 (GMI failure) lacked an egress across all ROIs of the gracilis muscle and lacked an ingress in the distal ROIs, whilst having a low overall fluorescence intensity.

The ROIs on the skin were placed as a positive control to indicate sufficient visualization after ICG injection, the influence of confounding variables and the reproducibility of the assessment. The skin perfusion curves had a similar shape to the muscle perfusion curve. The skin ROI of patient 3 had a particularly steep increase and high fluorescence intensity over time.

**Figure 4:**
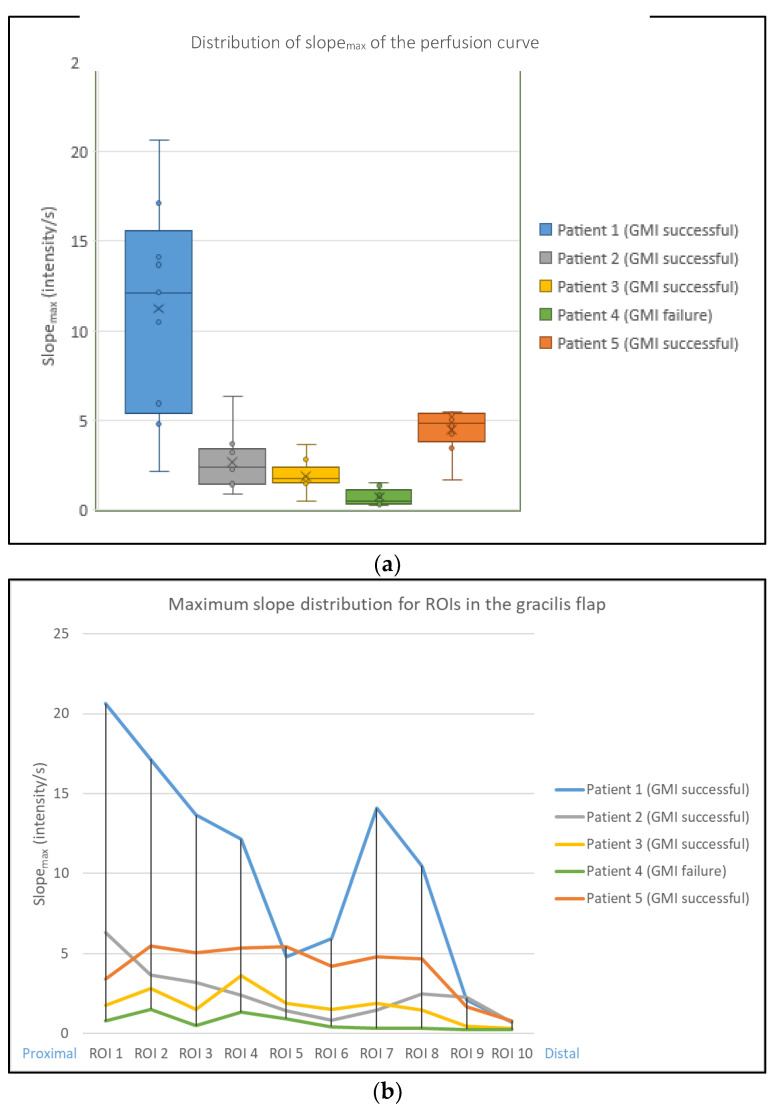
(**a**) The maximum slope of the curve for each ROI (slope_max_) was calculated and compared, including mean, maximum, minimum value and standard deviation. Slope_max_ was lowest in patient 4 (GMI failure). (**b**) The maximum slope distribution for ROIs 1 (proximal) to 10 (distal) was analyzed. Patient 1 showed a decrease across ROIs 5 and 6 and regained maximum slope distally. Patients 2, 3 and 5 showed a homogenous maximum slope distribution. Patient 4 (GMI failure) had the lowest maximum slope across the whole area of the gracilis muscle.

The maximum slope of the curve for each ROI (slope_max_) was calculated and compared, including its mean, maximum, minimum value, and standard deviation (Figure 4a). Slope_max_ was lowest in patient 4 (GMI failure). Slope_max_ was highest in patient 1 (GMI successful), as was the range between the maximum and minimum slope_max_ value.

The distribution of slope_max_ for ROIs 1–10 was analyzed for each patient (Figure 4b). Patient 1 showed a decrease across ROIs 5 and 6 and regained slope_max_ distally. Patients 2, 3 and 5 showed a homogenous slope_max_ distribution. Patient 4 (GMI failure) had the lowest slope_max_ distribution across all ROIs.

**Figure 5:**
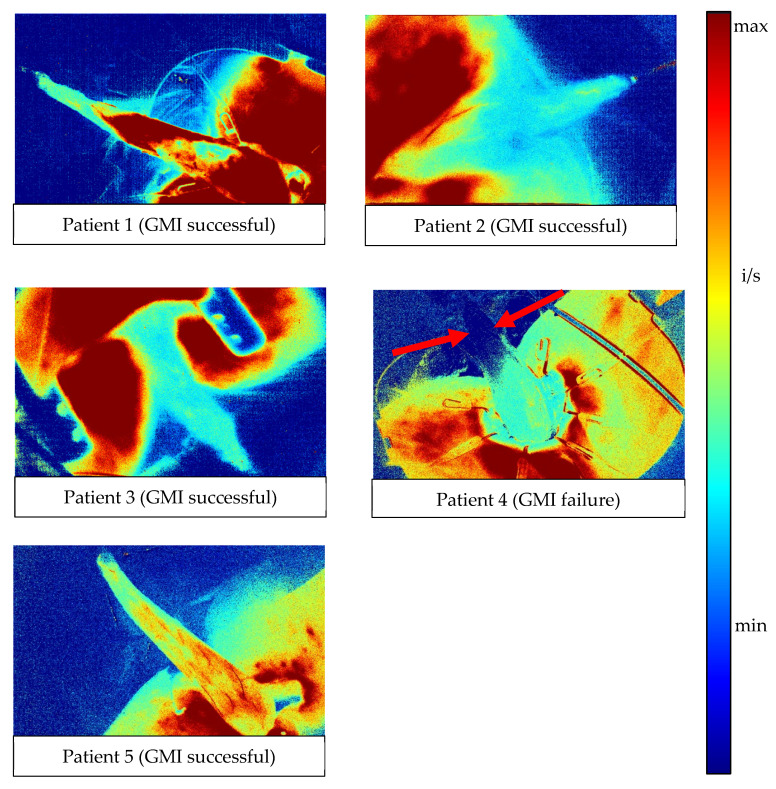
Automated pixel-to-pixel analysis of fluorescence intensity over time (patients 1–5). The maximum slope (slope_max_ value) per pixel was visualized by color-coding: red indicates high slope_max_ value, blue indicates no slope across the perfusion curve. In patient 4, only a small area near the base of the gracilis muscle shows a regional increase in the maximum slope. The distal, lateral, and dorsal parts of the muscle lack a slope of fluorescence intensity (dark blue), indicating poor blood flow in these areas (red arrows). In all other cases, there were higher slope_max_ values across the gracilis muscle (i/s: relative intensity units per second; min-max = minimum to maximum).

In the automated pixel-to-pixel analysis of the fluorescence intensity over time, the slope_max_ value per pixel was visualized by color-coding (Figure 5): red indicates a high slope_max_ value, and blue indicates no slope across the perfusion curve. Patients 1, 2, 3 and 5 had moderate to high slope_max_ values across the whole gracilis muscle (green/turquoise). In patient 4, only the proximal half of the gracilis muscle showed slope_max_ values. The distal half lacked slope_max_ completely (dark blue, red arrows).

**Figure 6:**
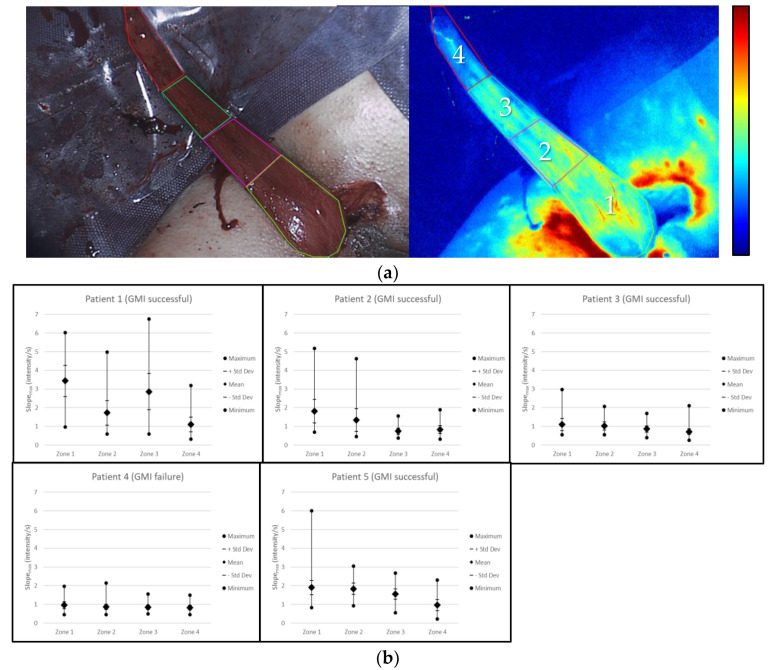
(**a**) Based on the anatomic direction of vascularization from the proximal base of the gracilis muscle to the distal tip, 4 perfusion zones (1–4) were determined (i/s: relative intensity units per second, min-max = minimum to maximum). (**b**) Scatter plot per patient: for perfusion zones 1–4, the distribution of the slope_max_ value per pixel was plotted, including the maximum, minimum and mean slope_max_ value and its standard deviation (SD).

Based on the anatomic direction of vascularization, perfusion zones 1–4 were determined (Figure 6a). By pixel-to-pixel analysis, the maximum, minimum and mean slope_max_ value and its standard deviation (SD) were calculated and plotted per zone (Figure 6b, scatter plot per patient).

Generally, across zones 1 (proximal)—4 (distal), there was a decrease in the range and mean slope_max_ value. In patients 1, 2, 3 and 5 (GMI successful), there was a greater range of slope_max_ values than in patient 4 (GMI failure). Patient 3 (GMI successful) and patient 4 (GMI failure) displayed lower mean slope_max_ values. However, in patient 3, the difference between the maximum and minimum value of slope_max_ was greater than in patient 4 across all zones, especially in zone 4 (distal).

## 4. Discussion

ICG fluorescence-based methods are gaining popularity for intraoperative perfusion visualization. This applies to plastic and reconstructive surgery, as well as general and colorectal surgery [19,22,23,24,25]. However, objective criteria for assessing the dynamic process of perfusion in tissues of surgical interest are still lacking. As reviewed before, the currently available devices and software solutions differ significantly from each other and often focus on the fluorescence intensity at a single point in time [17,18,26,27]. This may affect the validity and reliability of assessing perfusion. Slooter et al. performed an extensive review of quantitative fluorescence angiography methods, identifying fluorescence–time curves as the most promising future perfusion indicators [28]. The authors identified seven studies investigating fluorescence–time curves in humans, of which, five assess the clinical outcome of gastrointestinal anastomosis. While the studies describe a similar methodology, highlighting its potential, none of these assess muscle flap perfusion and its clinical outcome. In contrast to our results, while slope and several fluorescence time points are described, none of the mentioned studies identify slope_max_ or proceed to analyze its distribution, including a pixel-to-pixel analysis.

ICG-NIRF perfusion visualization for GMI and its software-based objective assessment have not previously been reported or correlated with the clinical outcome.

Using a comparatively low dose of ICG [29], an ICG-NIRF visualization of the gracilis muscle was achieved in all cases at the chosen time point with a subjectively sufficient fluorescence signal. 

However, in our data, the subjective parameters of visualization (signal strength, time to signal) did not show a correlation with the clinical outcome. This raises the question of whether an objective software-based perfusion assessment may be more valid.

The first step of our postoperative software-based assessment resulted in perfusion curves depicting fluorescence intensity over time for specifically determined ROIs (Figure 3). The pattern of an ingress followed by an egress could generally be identified for GMIs with a successful clinical outcome (patients 1, 2, 3 and 5). In contrast, patient 4 (GMI failure) showed no egress in the distal ROIs, suggesting impaired perfusion. The perfusion curves of patient 3 (GMI successful) show two ingress phases, with a more gradual flattening of the curve. The reason for this is unclear and a greater number of cases is required to investigate this topic further.

One ROI per patient was placed on the surrounding skin as a positive control (Figure 3). The perfusion curves of the control-ROIs indicated validity and a homogenous distribution of ICG after injection. They further suggested that the shape of ingress followed by egress is characteristic for several types of tissue.

The skin ROIs of patient 1 and 3 have a particularly steep increase and high fluorescence intensity over time. Future research into this is warranted.

When correlated with the clinical outcome, the perfusion curves confirm that the fluorescence intensity (the main marker in conventional, subjective ICG-NIRF assessment) may not be representative of perfusion, but rather the fluorescence intensity over time. The fluorescence intensity is generally lower in the distal part of the gracilis muscle, which can be explained anatomically by decreasing vascularization.

The identification of slope_max,_ and its distribution (Figure 4) supports these findings. The dip across ROIs 5 and 6 in the graph of patient 1 may be explained by superficial haematoma covering the muscle in that region (Figure 3, patient 1). The results propose that slope_max_ is a relevant indicator of perfusion in GMI, as it decreased across all regions of the gracilis muscle in the case of GMI failure (patient 4, Figure 4). This was confirmed by pixel-to-pixel and perfusion zone analysis (Figure 5 and Figure 6). The findings further suggest that the range between the minimum and maximum slope_max_ value across a tissue of interest may be more relevant to indicate impaired perfusion than the mean slope_max_ value (Figure 6). This could be explained by the presence of scattered blood vessels in areas of low vascularization, which increase the range of slope_max_ punctually despite a low mean slope_max_ value across that region (Figure 5 and Figure 6; patients 2 and 3).

The assessment steps presented here may lead to the identification of tissue-specific quantitative indicators of perfusion.

This study has some limitations. Only five patients were included. This number was insufficient for the statistical analysis of additional documented variables, such as haemodynamics, the distance of the camera to the surgical site, the subjective signal strength and the subjective time to signal. However, there seems to be no relevant influence of these variables on the fluorescence intensity, shape of the perfusion curve and slope_max_ value.

An intraoperative ICG-NIRF perfusion visualization was performed following a standardized protocol based on empirical single-center experience, which specified the ICG dosing and time of assessment and allowed for data collection during visualization. However, in one case (patient 4), there was clinical need for an additional perfusion visualization prior to the time point defined by the protocol, due to suspected pedicular impairment during mobilization. Here, the dose of ICG was increased for the second visualization. This highlights the need for standardized and objective protocols for the use of ICG-NIRF. In this case, the increased dose is unlikely to affect the postoperative software-based assessment, as this is based on the shape of the perfusion curve over time rather than on the interpretation of fluorescence intensity. These limitations need to be addressed in larger, prospective trials in the future, using real-time assessment software.

The presented pixel-to-pixel analysis (Figure 5) is a novel method of an automated perfusion assessment of the surgeon’s field of view. This method can visualize objective parameters, such as the fluorescence intensity over time and maximum or minimum slope under the perfusion curve.

The perfusion zones were determined to investigate different regions of vascularization (Figure 6). The perfusion zone analysis offers the possibility of determining perfusion zones of anatomic interest (such as zones of a muscle flap with decreasing vascularization from pedicle base to tip). As we can see by comparing Figure 3, Figure 4 and Figure 5 in correlation with the clinical outcome, the distribution of slopemax values across an anatomic area of interest may be more important to assess perfusion than fluorescence–time curves alone. Potentially, perfusion zone analysis can be performed for any individual surgical strategy and anatomic area of interest.

All aforementioned steps of the objective ICG-NIRF perfusion assessment can potentially be carried out in real-time, as the required time for calculation ranges from 1–5 min. We conducted this non-interventional retrospective study first in order to investigate its feasibility before altering procedures intraoperatively.

In this study, we present the feasibility of an ICG-NIRF perfusion visualization in GMI surgery. We further present, for the first time, a novel software-based multi-step approach for intraoperative real-time ICG-NIRF perfusion assessment and objective indicators of perfusion to potentially improve the clinical outcome in the future.

## Figures and Tables

**Table 1:** Clinical characteristics, indication, underlying condition and outcome of patients undergoing GMI from October 2019 to July 2020.

Patient No.	1	2	3	4	5
Indication for GMI	RVF	RVF	Perineal defect	PVF	PVF
Etiology	IC	CD	CD	UC	UC
Number of previous treatment attempts	3	2	1	2	1
Type of previous treatment	mucosal advancement flap with muscular plication	primary closure, endoluminal vacuum therapy	endoluminal vacuum therapy	primary closure, redo ileostomy	primary closure, redo ileostomy
Preoperative Medication	Adalimumab (paused)	Etoricoxib Azathioprine (paused)	Amlodipin Etoricoxib, Pregabalin, Buprenorphine	none	Certolizumab (paused), Etoricoxib
Age	52	29	42	52	37
BMI (kg/m^2^)	21.5	36.6	26.7	16.4	18.0
ASA	2	2	2	2	1
Clinical outcome within 3 months	GMI successful	GMI successful	GMI successful	GMI failure on day 43 after surgery	GMI successful
Revision Surgery within 3 months	No	No	No	Yes	No

Abbreviations: GMI = gracilis muscle interposition; RVF = rectovaginal fistula; PVF = pouch–vaginal fistula; IC = indeterminate colitis; CD = Crohn’s disease; UC = ulcerative colitis; BMI = body mass index; ASA = American Society of Anesthesiologists (ASA) score; (paused) = medication paused 3 weeks prior to surgery.

**Table 2:** Clinical and technical data collected at the time of intraoperative ICG-NIRF perfusion visualization.

Patient No.	1	2	3	4	5
Mean arterial blood pressure (mmHg)	72	70	78	69	78
Heart rate (beats/min)	62	67	54	80	77
Oxygen saturation (%)	99	99	100	98	99
Noradrenaline usage (µg/kg/min)	0.03	0	0	0.02	0
Time to fluorescence signal (s)	48	40	55	32	28
Subjective fluorescence signal strength (0–1–2)	2	1	1	2	2
Distance of camera to operating site (cm)	34	31	30	30	35

Abbreviations: mmHg = millimetre of mercury; min = minute; µg = micrograms; kg = kilograms; s = seconds; cm = centimeters subjective fluorescence signal strength: 0 = no signal, 1 = detectable signal, 2 = strong, homogenous signal.

## Data Availability

Not applicable.

## References

[1] Gilshtein H., Strassman V., Wexner S.D. (2020). Redo gracilis interposition for complex perineal fistulas. Tech. Coloproctol..

[2] Abu Gazala M., Wexner S.D. (2017). Management of rectovaginal fistulas and patient outcome. Expert Rev. Gastroenterol. Hepatol..

[3] Wilson T.R., Welbourn H., Stanley P., Hartley J.E. (2014). The success of rectus and gracilis muscle flaps in the treatment of chronic pelvic sepsis and persistent perineal sinus: A systematic review. Colorectal Dis. Off. J. Assoc. Coloproctol. Great Br. Irel..

[4] Singh M., Kinsley S., Huang A., Ricci J.A., Clancy T.E., Irani J., Goldberg J., Breen E., Bleday R., Talbot S.G. (2016). Gracilis Flap Reconstruction of the Perineum: An Outcomes Analysis. J. Am. Coll. Surg..

[5] Colibaseanu D.T., Dozois E.J., Walsh M.F. (2011). Amelioration of chronic pelvic sepsis secondary to a non-collapsible pelvic cavity using a gracilis rotational flap. Tech. Coloproctol..

[6] Wexner S.D., Ruiz D.E., Genua J., Nogueras J.J., Weiss E.G., Zmora O. (2008). Gracilis muscle interposition for the treatment of rectourethral, rectovaginal, and pouch-vaginal fistulas: Results in 53 patients. Ann. Surg..

[7] Samplaski M.K., Wood H.M., Lane B.R., Remzi F.H., Lucas A., Angermeier K.W. (2011). Functional and quality-of-life outcomes in patients undergoing transperineal repair with gracilis muscle interposition for complex rectourethral fistula. Urology.

[8] Zmora O., Potenti F.M., Wexner S.D., Pikarsky A.J., Efron J.E., Nogueras J.J., Pricolo V.E., Weiss E.G. (2003). Gracilis muscle transposition for iatrogenic rectourethral fistula. Ann. Surg..

[9] Hotouras A., Ribas Y., Zakeri S., Murphy J., Bhan C., Chan C.L. (2015). Gracilis muscle interposition for rectovaginal and anovaginal fistula repair: A systematic literature review. Colorectal Dis. Off. J. Assoc. Coloproctol. Great Br. Irel..

[10] Pinto R.A., Peterson T.V., Shawki S., Davila G.W., Wexner S.D. (2010). Are there predictors of outcome following rectovaginal fistula repair?. Dis. Colon Rectum.

[11] Maeda Y., Heyckendorff-Diebold T., Tei T.M., Lundby L., Buntzen S. (2011). Gracilis muscle transposition for complex fistula and persistent nonhealing sinus in perianal Crohn’s disease. Inflamm. Bowel Dis..

[12] Fürst A., Schmidbauer C., Swol-Ben J., Iesalnieks I., Schwandner O., Agha A. (2008). Gracilis transposition for repair of recurrent anovaginal and rectovaginal fistulas in Crohn’s disease. Int. J. Colorectal Dis..

[13] Rottoli M., Vallicelli C., Boschi L., Cipriani R., Poggioli G. (2018). Gracilis muscle transposition for the treatment of recurrent rectovaginal and pouch-vaginal fistula: Is Crohn’s disease a risk factor for failure? A prospective cohort study. Updates Surg..

[14] Takano S., Boutros M., Wexner S.D. (2014). Gracilis transposition for complex perineal fistulas: Rectovaginal fistula and rectourethral fistula. Dis. Colon Rectum.

[15] Korsun S., Liebig-Hoerl G., Fuerst A. (2019). Gracilis muscle transposition for treatment of recurrent anovaginal, rectovaginal, rectourethral, and pouch-vaginal fistulas in patients with inflammatory bowel disease. Tech. Coloproctol..

[16] Chwei-Chin Chuang D., Wei F.-C., Mardini S. (2009). CHAPTER 29—Gracilis flap. Flaps and Reconstructive Surgery.

[17] Khavanin N., Qiu C., Darrach H., Kraenzlin F., Kokosis G., Han T., Sacks J.M. (2019). Intraoperative Perfusion Assessment in Mastectomy Skin Flaps: How Close are We to Preventing Complications?. J. Reconstr. Microsurg..

[18] Lütken C.D., Achiam M.P., Osterkamp J., Svendsen M.B., Nerup N. (2020). Quantification of fluorescence angiography: Toward a reliable intraoperative assessment of tissue perfusion—A narrative review. Langenbeck’s Arch. Surg..

[19] van den Bos J., Al-Taher M., Schols R.M., van Kuijk S., Bouvy N.D., Stassen L.P.S. (2018). Near-Infrared Fluorescence Imaging for Real-Time Intraoperative Guidance in Anastomotic Colorectal Surgery: A Systematic Review of Literature. J. Laparoendosc. Adv. Surg. Tech. A.

[20] Meijer D.K.F., Weert B., Vermeer G.A. (1988). Pharmacokinetics of biliary excretion in man. VI. Indocyanine green. Eur. J. Clin. Pharmacol..

[21] Nerup N., Andersen H.S., Ambrus R., Strandby R.B., Svendsen M.B.S., Madsen M.H., Svendsen L.B., Achiam M.P. (2017). Quantification of fluorescence angiography in a porcine model. Langenbeck’s Arch. Surg..

[22] Li K., Zhang Z., Nicoli F., D’Ambrosia C., Xi W., Lazzeri D., Feng S., Su W., Li H., Ciudad P. (2018). Application of Indocyanine Green in Flap Surgery: A Systematic Review. J. Reconstr. Microsurg..

[23] van den Bos J., Jongen A., Melenhorst J., Breukink S.O., Lenaerts K., Schols R.M., Bouvy N.D., Stassen L.P.S. (2019). Near-infrared fluorescence image-guidance in anastomotic colorectal cancer surgery and its relation to serum markers of anastomotic leakage: A clinical pilot study. Surg. Endosc..

[24] Mangano A., Fernandes E., Gheza F., Bustos R., Chen L.L., Masrur M., Giulianotti P.C. (2019). Near-Infrared Indocyanine Green-Enhanced Fluorescence and Evaluation of the Bowel Microperfusion During Robotic Colorectal Surgery: A Retrospective Original Paper. Surg. Technol. Int..

[25] Blanco-Colino R., Espin-Basany E. (2018). Intraoperative use of ICG fluorescence imaging to reduce the risk of anastomotic leakage in colorectal surgery: A systematic review and meta-analysis. Tech. Coloproctol..

[26] Sood M., Glat P. (2013). Potential of the SPY intraoperative perfusion assessment system to reduce ischemic complications in immediate postmastectomy breast reconstruction. Ann. Surg. Innov. Res..

[27] Griffiths M., Chae M.P., Rozen W.M. (2016). Indocyanine green-based fluorescent angiography in breast reconstruction. Gland. Surg..

[28] Slooter M.D., Mansvelders M.S.E., Bloemen P.R., Gisbertz S.S., Bemelman W.A., Tanis P.J., Hompes R., van Berge Henegouwen M.I., de Bruin D.M. (2021). Defining indocyanine green fluorescence to assess anastomotic perfusion during gastrointestinal surgery: Systematic review. BJS Open.

[29] Pruimboom T., van Kuijk S.M.J., Qiu S.S., van den Bos J., Wieringa F.P., van der Hulst R., Schols R.M. (2020). Optimizing Indocyanine Green Fluorescence Angiography in Reconstructive Flap Surgery: A Systematic Review and Ex Vivo Experiments. Surg. Innov..

